# Microscale Bioreactors for *in situ* characterization of GI epithelial cell physiology

**DOI:** 10.1038/s41598-017-12984-2

**Published:** 2017-10-02

**Authors:** Cait M. Costello, Mikkel B. Phillipsen, Leonard M. Hartmanis, Marek A. Kwasnica, Victor Chen, David Hackam, Matthew W. Chang, William E. Bentley, John C. March

**Affiliations:** 1 Department of Biological and Environmental Engineering, Cornell University, Ithaca, USA; 2 Division of Pediatric Surgery, Department of Surgery, Johns Hopkins University, Baltimore, USA; 3 Department of Biochemistry, Yong Loo Lin School of Medicine, NUS, Singapore, Singapore; 4 Institute for Biomedical Devices, University of Maryland, Maryland, USA

## Abstract

The development of *in vitro* artificial small intestines that realistically mimic *in vivo* systems will enable vast improvement of our understanding of the human gut and its impact on human health. Synthetic *in vitro* models can control specific parameters, including (but not limited to) cell types, fluid flow, nutrient profiles and gaseous exchange. They are also “open” systems, enabling access to chemical and physiological information. In this work, we demonstrate the importance of gut surface topography and fluid flow dynamics which are shown to impact epithelial cell growth, proliferation and intestinal cell function. We have constructed a small intestinal bioreactor using 3-D printing and polymeric scaffolds that mimic the 3-D topography of the intestine and its fluid flow. Our results indicate that TEER measurements, which are typically high in static 2-D Transwell apparatuses, is lower in the presence of liquid sheer and 3-D topography compared to a flat scaffold and static conditions. There was also increased cell proliferation and discovered localized regions of elevated apoptosis, specifically at the tips of the villi, where there is highest sheer. Similarly, glucose was actively transported (as opposed to passive) and at higher rates under flow.

## Introduction

Immortal intestinal epithelial cell lines such as Caco-2 are useful for *in vitro* studies of intestinal function, as they have ability to form polarized monolayers on membranes that separate the apical and basolateral space. This is important since the intestine’s primary function is digestion and absorption, and there are many studies of the intestine that require monitoring of molecules through the epithelial cell layer from the apical to the basolateral compartments and vice versa. Transwells® have long been used as the standard *in vitro* culture method for studies of intestinal absorption, as they provide both an apical and basolateral spaces to simulate the gut-blood-barrier and enable both active and passive transport of drugs and nutrients^1,2^. However, intestinal cells seeded onto flat supports exhibit markedly different phenotypes to cells *in vivo* ^3^, partly due to the poor representation of the 3-D extracellular microenvironments. We have previously shown that recreating the topography of the small intestine with biocompatible collagen or poly-lactic-glycolic acid (PLGA) scaffolds populated with accurately sized villi can lead to improved differentiation and paracellular permeability of Caco-2 monolayers along the villus axis^4–6^. Cells in a 3-D villus environment experience different nutrient gradients (including oxygen) than cells grown on flat surfaces^7^, which can affect their physiology including differentiation. Also, spatial microstructure can influence cell-cell junctions, cell-matrix contacts and molecular communication^8,9^.

In addition to the complex topographical and cellular environment, the human intestine exhibits mechanically active peristaltic motions and fluid flow after ingestion that guide the food bolus down the intestine, facilitating absorption. Specifically, the stomach and small intestine create shear stresses via the hormone-mediated migrating-motor-complex (MMC), an inter-digestive pattern which is dominated by cycles of stasis and short, high-pressure bursts of peristaltic motions, that serve to propel small particles and microorganisms towards the colon^10–12^. Even during fasting, there is an intermittent flow that exposes the intestinal epithelia to shear stresses. Cyclic strain and shear stress have been shown to modulate signaling pathways in the gut, including those mediated by mechano-sensing β1 and β3 integrins. Importantly, these activate the Rac1 and ERK-signaling pathways (among others) and the downstream wnt/β-catenin pathways which control cellular proliferation and differentiation^13–17^.

On-chip (*in vitro*) models have paved the way for studies of intestinal epithelia and/or bacteria in conjunction with fluid flow^18–22^. Some have replicated mechanical stimuli and shear stresses created by fluid flow and revealed altered cell differentiation and morphology, adhesion, and paracellular permeability^20,23,24^ more representative of the human system. Often dual chambers split the model into an apical (upper) and basolateral (lower) compartments, simulating the gut-blood-barrier. They also provide fluid ports to enable gas and nutrient exchange. For example, Kim *et al*.^20^ created a “gut on chip” device that enabled the co-culture of Caco-2 cells and *Lactobacillus rhamnosus* on a porous membrane, with fluid flow channels for sustained cell culture, and cyclic strain for mimicking peristaltic motions. They found that cyclic strain caused an increase in Caco-2 elongation, Transepithelial electrical resistance (TEER), differentiation and paracellular permeability. In a similar fashion, Giusti *et al*. found that Caco-2 cells grown in a microfluidic device had greater expression of tight junctions and a threefold increase in fluorescein permeability compared to static controls^25^. Shah *et al*. created a multichamber device to monitor crosstalk between bacterial species and the epithelial layer, with the added ability to sample oxygen concentrations to map the formation of facultative anaerobe microbial niche^26^.

What is missing is a device that provides both the accurately-sized villus topography and fluid flow to improve study of intestinal absorption, drug delivery, and intestinal barrier function. Towards this aim, we developed a 3-D printed bioreactor that can both contain villus scaffolds and also create separation of the apical and basolateral spaces in a manner in which fluid flow exposes intestinal epithelial cells to physiologically relevant shear stresses.

## Results

### Bioreactor assembly

**Figure 1:**
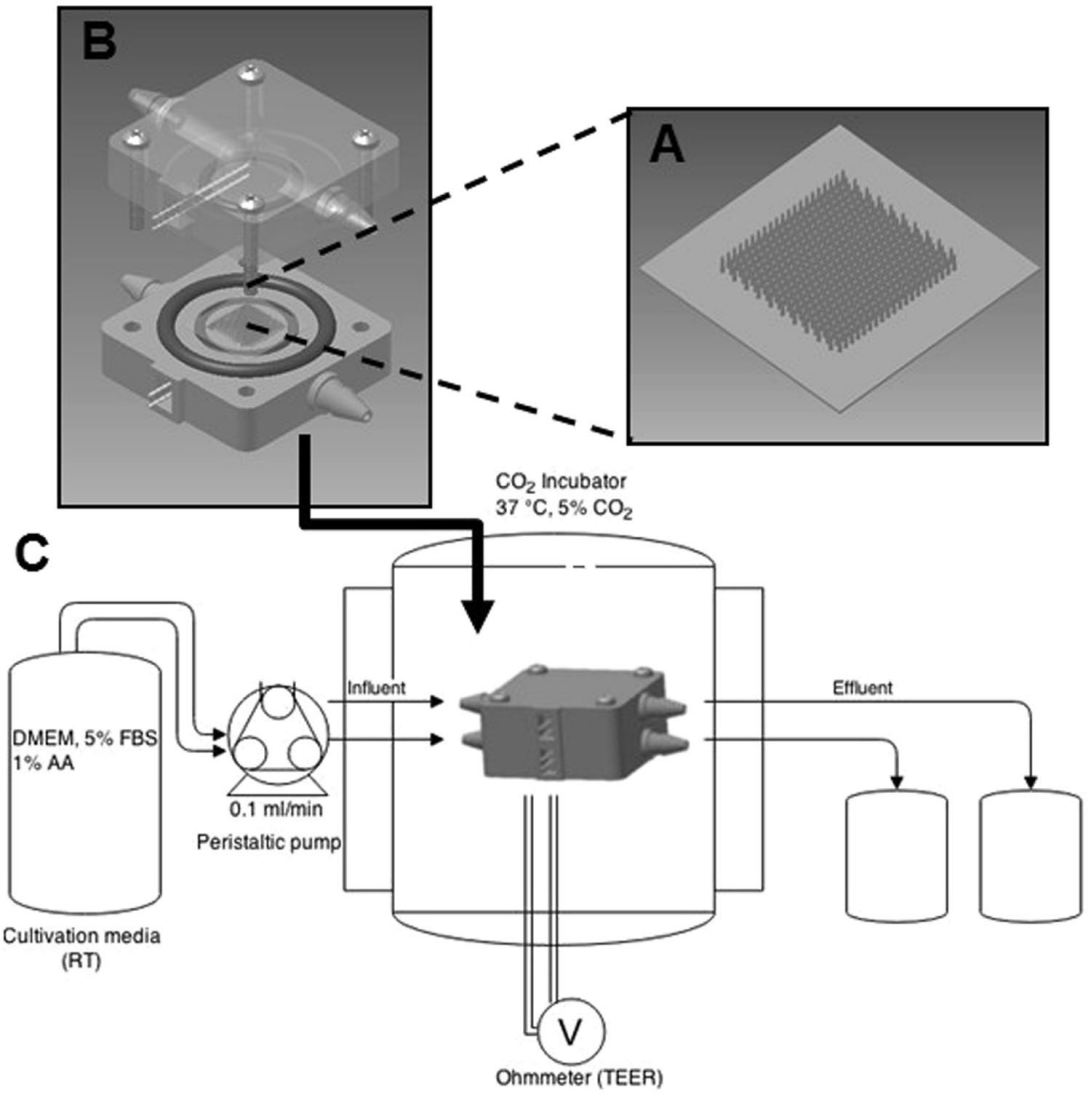
Bioreactor Setup. A porous PEVA scaffold (**A**) was seeded with Caco-2 cells and fitted into the assembled 3-D printed bioreactor vessel (**B**) using the o-rings to seal it in place. After connection to influent and effluent tubing and the peristaltic pump, the device is placed inside the CO_2_ incubator for 3–5 weeks (**C**). TEER measurements were taken daily by connecting the silver wires to chopstick electrodes and then to a voltohmmeter.

To replicate the topography of the intestine, we constructed porous villous scaffolds via micromolding as described previously, however we substituted our previous material (PLGA) with poly-ethylene-co-vinyl-acetate (PEVA) as it is not biodegradable, and therefore is likely to be more resistant to erosion by shear. In order to combine the 3-D topography of the intestine with apical and basolateral flow fields, we inserted our PEVA scaffolds into a fluidic device to form a small intestinal bioreactor. The bioreactors were designed using AutoCAD Inventor® and fabricated via 3-D printing in an Object30 Pro 3D-printer, using VeroClear-RGD810 as the source material. A schematic representation of the bioreactor setup is shown in Fig. 1. The device consists of top and bottom cassette holders designed to enclose a 10 × 10 mm GI cassette, forming apical and basolateral spaces above and below; two fluidic connectors are included on opposite ends of the bottom holder and four 2 mm diameter holes provide for screws that clamp the holders in place. Also, two sockets are provided on top and bottom for o-rings that prevent leaking when assembled. The o-rings also separate the scaffold on each side to ensure that transfer occurs only through the epithelial monolayer and the scaffold itself, allowing for studies of absorption. In addition, we added two 0.55 mm diameter channels to thread silver wire through to the apical and basolateral space, which can connect to a voltohmmeter for TEER measurements. The influent tubes connect the reactors to a peristaltic pump to continually flow media at the desired velocity, and effluent media bottles enable sampling of passive or actively transported nutrients/drugs from the apical to basolateral space. All printed parts were coated with Paralene® by vapor deposition prior to assembly, which protects the device from erosion by the media and provides a biocompatible, water-tight layer to protect the cells from leaching from the device parts. For controls in static conditions, we integrated our PEVA scaffolds into a custom insert kit as described previously^5,6^.

### Shear Stress

**Figure 2:**
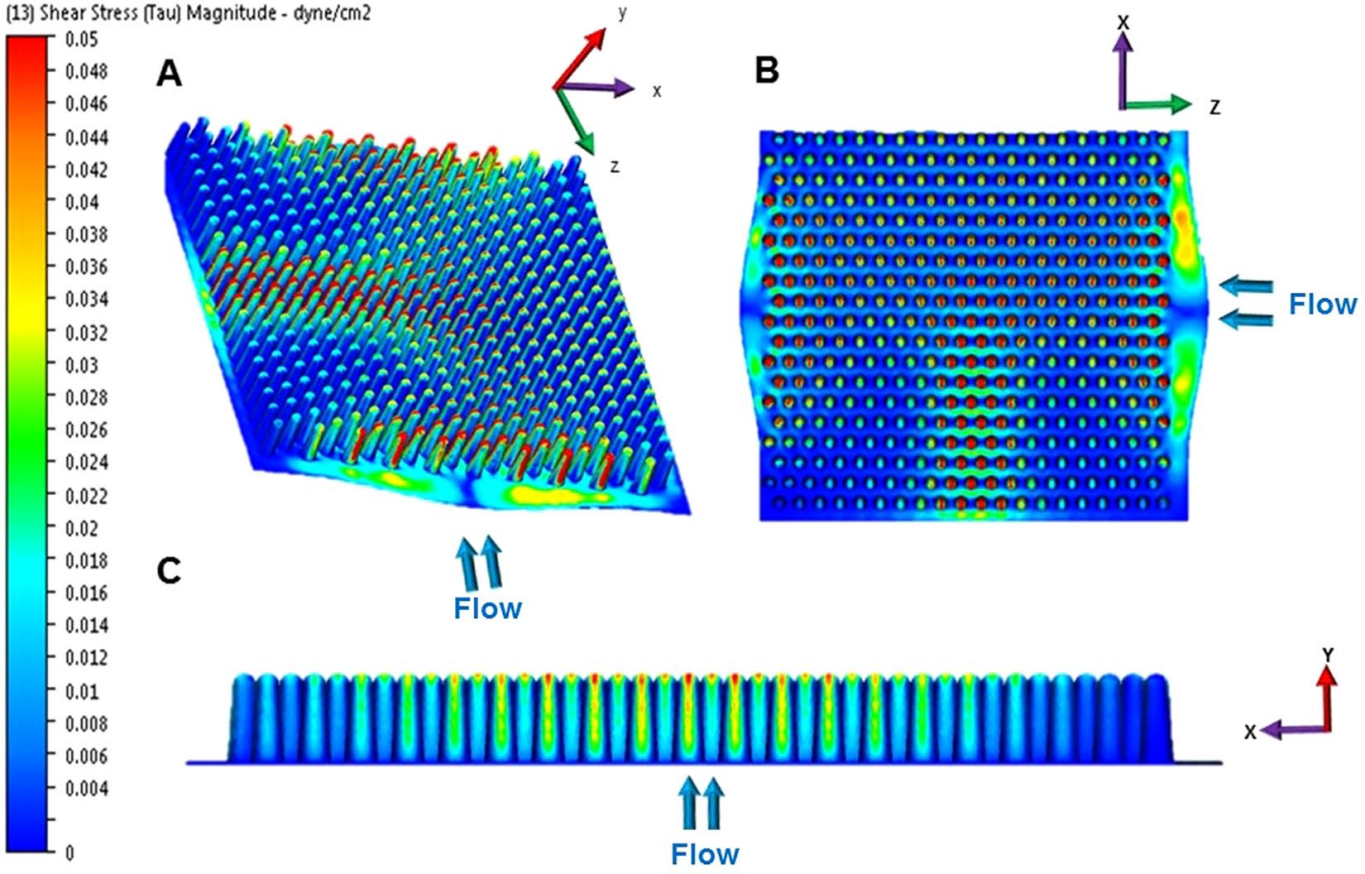
Computational fluid dynamics (CFD) with intestinally relevant flow rates resulting in shear stress within the bioreactor between 0.002 and 0.1 dyne/cm^2^. The CFD images show different angles of the scaffold, with a 3D profile (**A**) top down view (**B**) and planes that were cut through the middle of the scaffold in the and x-y (**C**) directions. Blue arrows indicate direction of flow. High shear (red) is dominated mainly at the tips of the villi, and in between the villi we have levels of intermediate shear with mainly low shear at the base. Note: these images also include the TEER wires (not shown) which cause a ‘strip’ of intermediate shear in the middle of the scaffolds.

For simplicity and reproducibility, we chose to simulate the baseline MMC of a fasting healthy human, which occurs in between feeding and is characterized by a wave of contractions that passes along the intestine for 6–10 mins every 80–100 mins^27^. We chose a median flow rate of 8 minutes ‘contraction’ or pulse of flow, with a period of stasis for 90 minutes with no flow, which was repeated in an on/off cycle. In order to simulate the flow fields in our reactors, we used computational fluid dynamics (CFD) with to-scale computer aided design (CAD) drawings of the bioreactor, scaffold, and 20 µm epithelial layer. Our results indicated shear stress within the reactor between 0.001 and 0.05 mdyne/cm^2^ (Fig. 2). Results indicate that the area around the base of the intestinal scaffolds is essentially shielded from high shear while the areas at the tips of the villi experience orders of magnitude higher shear rates. Further, within the scaffold field (that is, between the villi) there is a gradient of shear. From these simulations we are able to see that there are shear gradients in two directions: *along the villus axis* (C, y-direction, in Fig. 2) *and within the villus field* (A and B, x & z direction in Fig. 2).

### TEER and Tight Junction Formation

**Figure 3:**
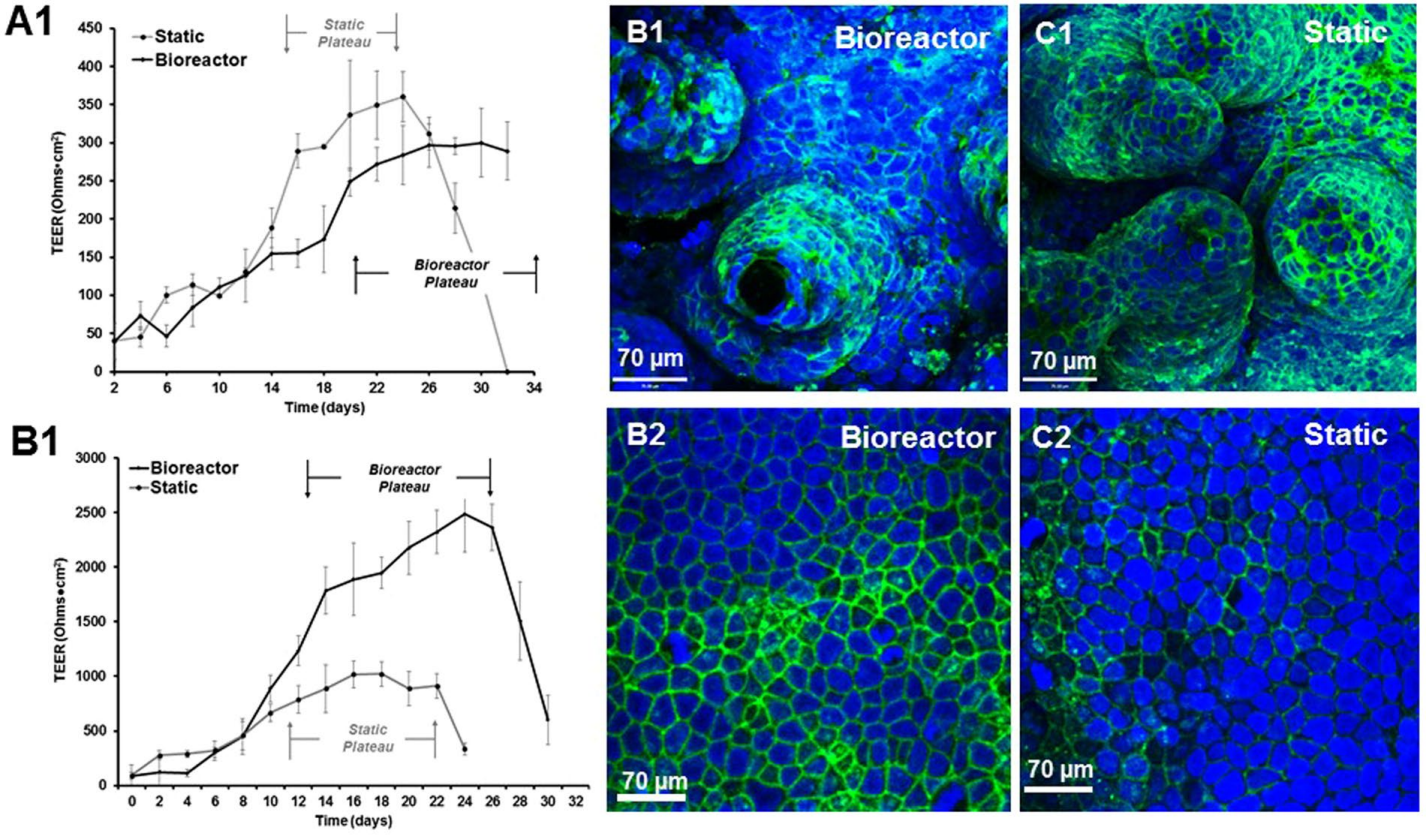
TEER measurements of Caco-2 monolayers over 32 days in the bioreactors and under static conditions (N = 6), on flat scaffolds and in 3-D scaffolds. For 3-D scaffolds, in both conditions, TEER increases steadily over the course of the culture until a peak or ‘plateau’ is reached, which is typically representative of an intact barrier for absorption studies (**A1**). The plateau takes longer to reach in the bioreactors, but it is also maintained for almost two weeks; in contrast in the static conditions the plateau lasts for one week. With flat scaffolds, TEER increases in the bioreactor by approximately 10 fold. TEER also increases in the static reactors, but at a lower magnitude than in bioreactors (**B1**). Scaffolds were also stained for tight junctions with Claudin-1 (**B**,**C**). 3-D bioreactor scaffolds have less staining at the base than static scaffolds, and conversely flat bioreactor scaffolds have more staining than the static scaffolds.

Using silver wires attached to chopstick electrodes, we monitored TEER over the course of 32 days, on both 3-D scaffolds and flat scaffolds. For 3-D scaffolds, the results show that TEER peaked around day 16-17 in static conditions, and then the monolayer started to break down around day 24-25 (Fig. 3A1). However, in the bioreactor, the TEER experienced a delay in plateau with a peak around day 20 and was able to sustain the monolayer for up to 5 weeks. (Confocal images at 5 weeks can be found in Supplementary Fig. 1). In contrast, on a flat scaffold, perfusion of flow causes a dramatic increase in TEER compared to static conditions, almost 10 fold higher. Additionally, we were not able to maintain the monolayer beyond the 32 day period. We supported our TEER data by staining for tight junction protein, Claudin-1 (Fig. 3B,C). In the 3-D scaffolds, there are significantly reduced tight junctions at the base of the scaffold compared to static where there is heavy staining everywhere (image analysis shown in Supplementary Fig. 2). In the flat scaffolds, the reverse is true; there is greater staining for tight junction proteins in the bioreactor than in the static conditions.

### Markers of Differentiation

**Figure 4:**
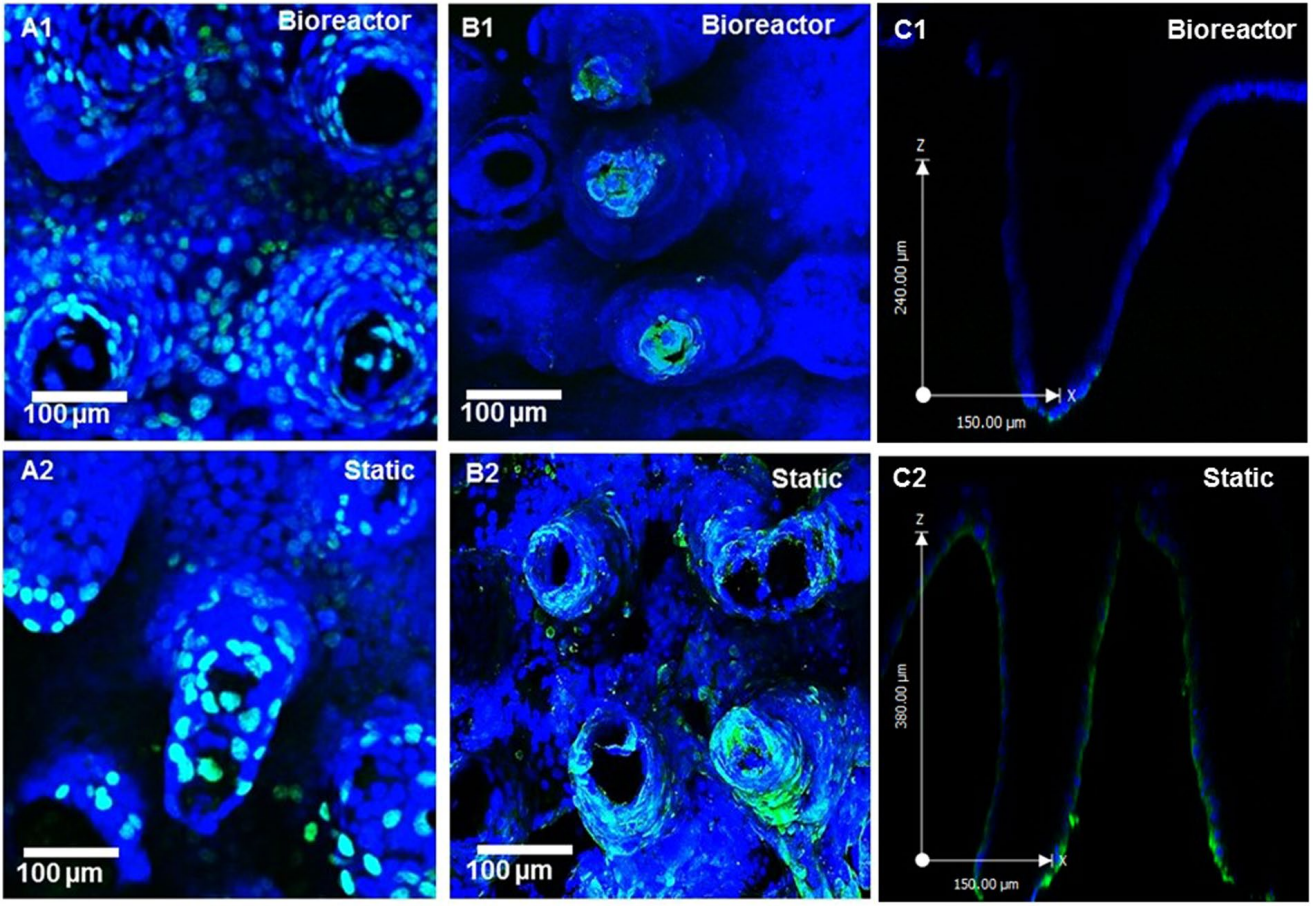
3D confocal rendering of PEVA scaffolds after growth of Caco-2 cells in the bioreactors and under static conditions. Images were taken at 20X magnification and stained for proliferation with EdU staining (green) (**A1-2**) and apoptosis with Tunel Assay (green) (**B1**-**2**). Caco-2 monolayers grown in the bioreactors have more proliferating cells and the apoptotic cells are localized to the tips of the villi where the highest shear is. Conversely, the static monolayers have less proliferating cells near the base and a large increase in apoptotic cells. A cross-section of the scaffolds show that apoptosis (green) is limited to the tips of the villi under flow conditions, and conversely is along the length of the villi in static conditions (**C1-2**).

**Figure 5:**
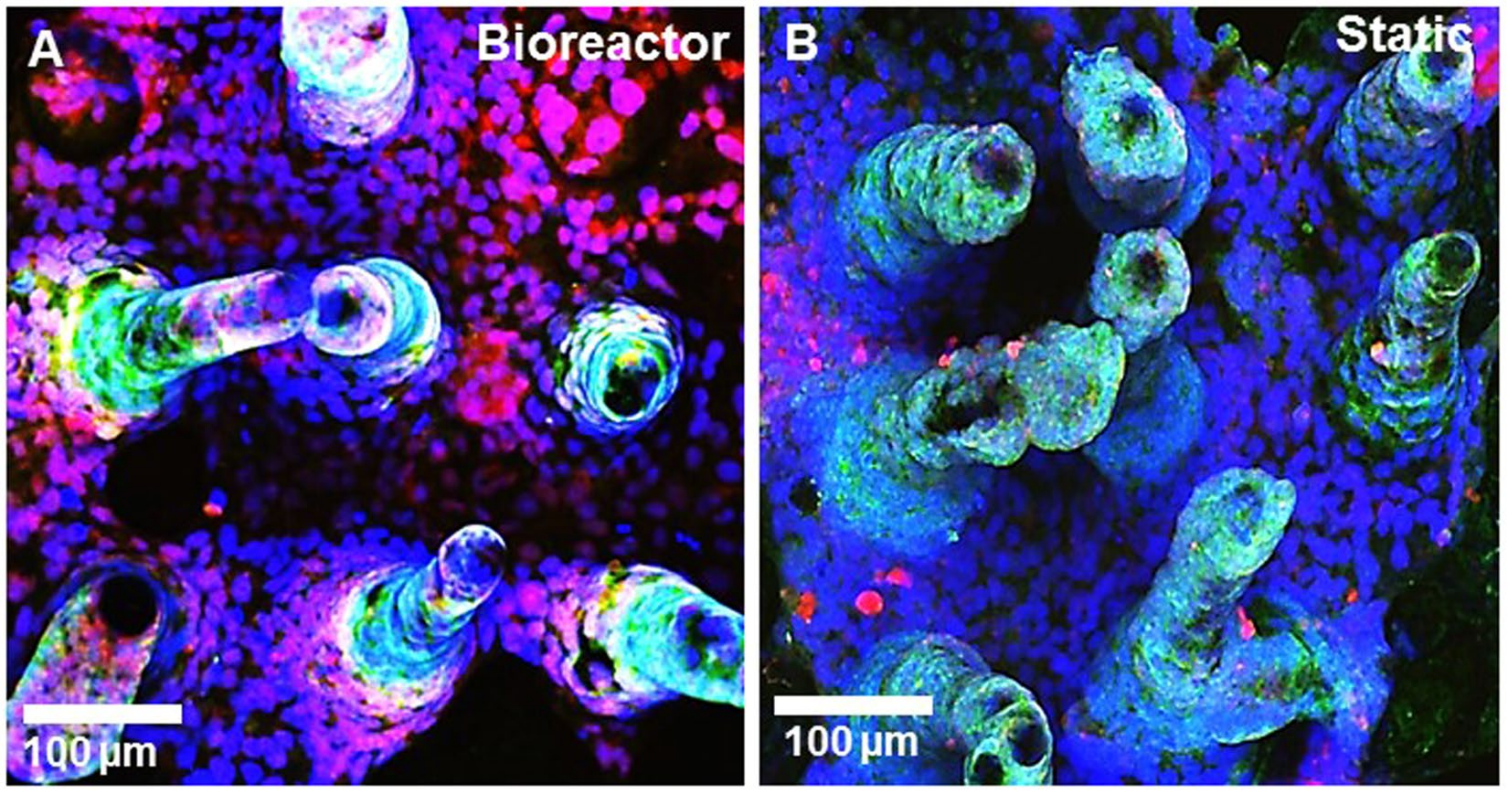
3D confocal rendering of PEVA scaffolds after growth of Caco-2 cells in the bioreactors (**A**) and under static conditions (**B**). Images were taken at 20X magnification. Caco-2 were stained for alkaline phosphatase with goat anti-alkaline phosphatase (green) and mucus with WGA (red). Caco-2 monolayers grown in the bioreactors have less alkaline phosphatase near the base but a dramatic increase in the amount of mucus.

3-D scaffolds were stained for markers of proliferation via EdU incorporation, and apoptosis via Tunel assay. We saw a greater number of proliferative cells in the bioreactor all along the length of the villi and to the base, whereas static scaffolds mainly had proliferative cells at the tips, as shown by the Edu incorporation in Fig. 4A1-2. Conversely, bioreactor scaffolds showed remarkably localized apoptosis at the tips of the villi by using the Tunel Assay to detect apoptotic cells, whereas the static scaffolds stained for apoptosis all along the length of the villi (Fig. B1-2, C1-2). Spatial distribution of apoptosis along the length of the villi was determined via image analysis (Supplementary Fig. 3) and found to be significantly higher at the base of the scaffolds under static conditions compared to flow. Scaffolds were also stained for alkaline phosphatase and mucus (Fig. 5). Both under flow and under static conditions, both scaffolds had significantly higher alkaline phosphatase at the tips than at the base (Supplementary Fig. 4). The static scaffolds has slightly reduced alkaline phosphatase at the base compared to static, however this was not significant after image analysis. Mucus production via staining with WGA-lectin was dramatically increased in the flow conditions compared to static.

### Active Transport of Glucose

**Figure 6:**
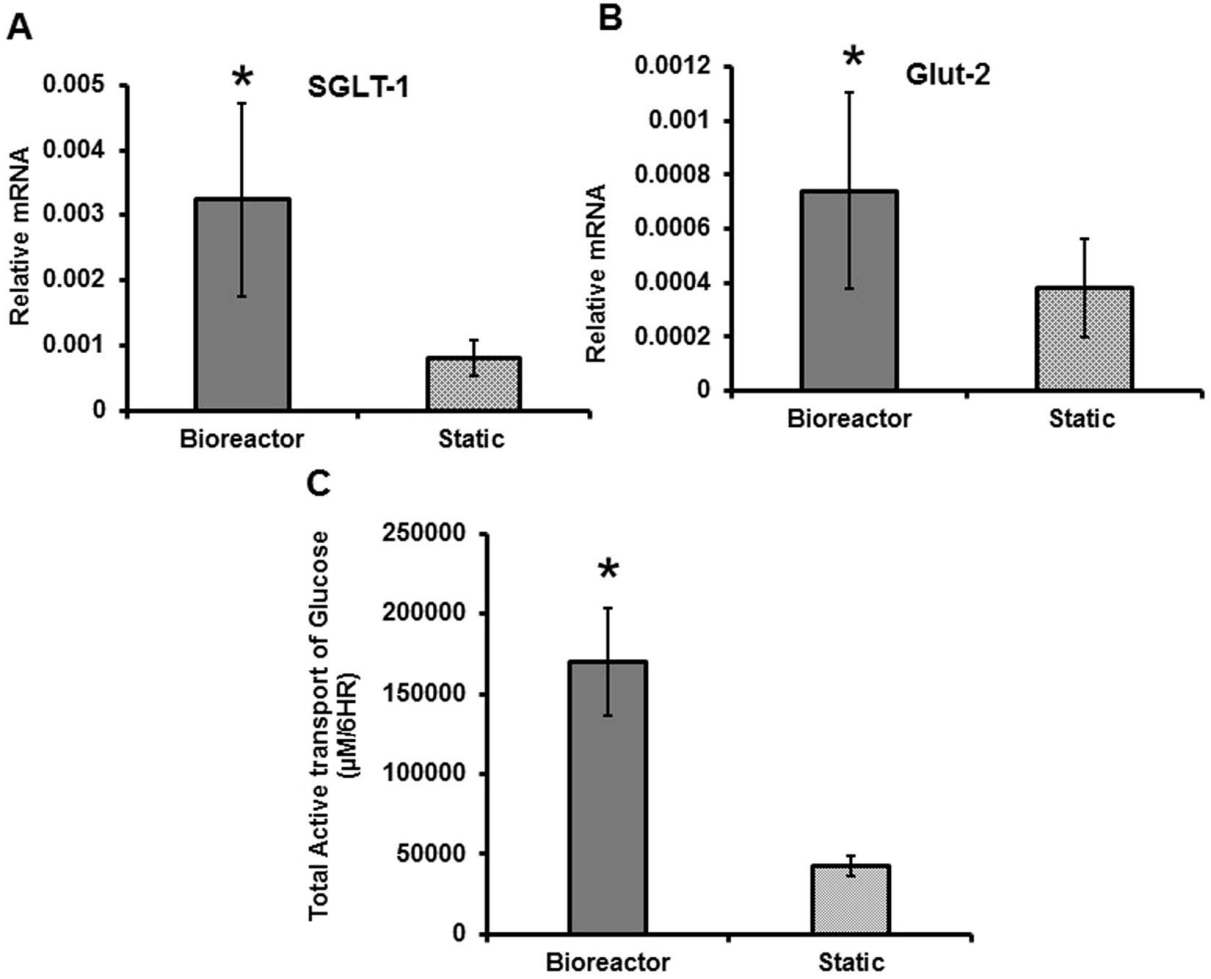
Relative mRNA levels of glucose transporters SGLT-1 (**A**) and Glut-2 (**B**) in Caco-2 grown in bioreactors under shear stress and control scaffolds under static conditions. Significance was determined by a Student’s t-test. N = 6. Active transport of glucose over 6 hours in the bioreactors and static scaffolds (**C**). For statistical differences, the area under the curve (AUC) was calculated for each sample and then significance was determined using a Student’s t-test (B). N = 3.

We measured levels of mRNA for two glucose transporters found in intestinal epithelium, SGLT-1 and Glut-2 via qPCR. Figure 6A,B shows that after 3 weeks in the bioreactor, the mRNA levels of SGLT-1 and Glut-2 were three-fold and two-fold higher than in the static conditions respectively, and these results were significant as determined by a Student’s t-test. We measured active transport of glucose by luminally feeding the cells glucose under perfusion, and then measuring concentration of glucose that passed through the monolayer, by sampling basolaterally at time intervals for analysis with a glucose assay. We then repeated this study by blocking the glucose transporters, using Phlorizin and Phloretin, so that we could determine the amount of passive diffusion of glucose through the monolayer, and use this to calculate the active transport. As shown in Fig. 6C, Caco-2 monolayers under flow conditions significantly actively transported more glucose than the static systems over a period of 6 hours. The raw data for this study is shown in Supplementary Fig. 4.

## Discussion

In our previous studies we used PLGA as the villus and membrane scaffolding material as it is biocompatible and biodegradable and can be used for reconstructive surgical implants^28,29^. However, for long-term *in vitro* studies under flow conditions as desired here, a scaffolding material that would not be eroded by shear stress would be more useful. We used PEVA here, as it is not biodegradable and has been used successfully for tissue culture *in vivo* ^30,31^ and *in vitro* ^32^. Importantly, this material can be easily substituted as we can exploit the same solvents and molding techniques that we employed previously^6^. In addition, PEVA has a lower young’s modulus/elasticity than PLGA (~0.03 GPa^33^ versus ~2.0 GPa^34^ respectively); we anticipated this would enable a degree of scaffold villi displacement from their position, like actual villi. Humans are intermittent feeders, and as such in the real intestine the shear stress levels are not constant; levels of high and low shear stress are dominated by periods of fasting and feeding. Levels of shear stress *in vivo* are dictated by peristaltic segmentation, consequently shear rates are subject to variations in ingestion of food. We note that reports of flow dynamics in simulations and in animals are highly varied^20,23,25,35,36^, and to simplify our experiments we chose to just simulate a single flow rate.

The typical set-up for absorption/permeability experiments using Caco-2 monolayers is to culture on Transwells® for 15–21 days, during which cells proliferate, become confluent and then differentiate to form tight junctions and express brush border enzymes^37^. We have previously shown that culturing Caco-2 on villus scaffolds causes a reduction in TEER and an increase in brush border enzyme expression compared to Transwells®. To assess the effect of flow we chose to follow the same culture set-up, by growing the cells for up to 32 days in the reactors under flow, using scaffolds cultured under static conditions as a control. Measuring TEER in Caco-2 monolayers enables tracking of the formation of tight junctions and barrier function in real time over the course of the cell culture. Typically, cells cultivated on Transwells® exhibit a steady increase in TEER which then levels off after 14–15 days as cells reach confluence and form tight junctions. Other studies with similar flow devices have shown that flat monolayers of Caco-2 under perfusion actually increase in TEER compared to static^20,36^ and also reach maximal TEER quicker than static conditions^38^ as shear stress is positively correlated with an increase in cellular differentiation. Similarly, we found that TEER was greater in the bioreactor than in static on a flat scaffold, in contrast to being lower in a villous scaffold. On a flat scaffold, the monolayer is exposed to shear in all areas, which may cause the monolayer to grow at an expedited rate.

However, on a 3-D scaffold, we saw a reduction in TEER and reduction in tight junction staining. We should note that we also see a lag that is unusual for TEER growth curves, and we attribute this delay in plateau due to the fact that we seeded directly into the scaffolds, and the initial perfusion may cause detachment to an unestablished monolayer. In our preliminary studies, we grew scaffolds for one week in static conditions and we did not see a delay in TEER (data not shown). We note that *ex vivo*, human intestine TEER values are often much lower^39^ indicating leakier monolayers. In our CFD simulations, we showed that the base of the villi if protected from shear and there is much higher shear at the tips. Phenomenologically, *in vivo* cell sloughing occurs from the tips of the villi, and this is compensated for by proliferating cells migrating up the villi to replace the lost cells. The cell turnover in the native intestine is typically 4–5 days. Studies have shown that fully differentiated intestinal epithelial cells are more likely to experience anoikis (a loss of adhesion) which is associated with a loss of integrin function at the tips of the villi^40–42^. It is possible that in the bioreactors, exposure of Caco-2 to high shear at the tips of the villi causes accelerated differentiation of the cells at the tips and, in turn, more anoikis. This coupled with the force of the flow may lead to increased cell shedding which could provide more room for the cells underneath to proliferate and replace the cells above. This is supported by the fact that in our TEER study with a flat scaffold, we were not able to maintain the monolayer beyond 32 days, whereas the bioreactors can grow for at least 5 weeks, possibly due to the fact that monolayer likely would not reach the same level of confluence or form multilayers as in a Transwell or a flat device.

This hypothesis is supported by staining the scaffolds for markers of proliferation and apoptosis (Fig. 4). That is, we saw a greater number of proliferative cells in the bioreactor all along the length of the villi and to the base, whereas static scaffolds mainly had proliferative cells at the tips, as shown by the Edu incorporation in Fig. 4A1-2. Conversely, bioreactor scaffolds showed remarkably localized apoptosis at the tips of the villi by using the Tunel Assay to detect apoptotic cells, whereas the static scaffolds stained for apoptosis all along the length of the villi (Fig. B1–2). The bioreactors likely provide conditions for the monolayer to continually recycle, whereas we would eventually have monolayer breakdown and mass cell lift off on the static scaffolds. Another of the differences between current *in vitro* intestinal models and live animals is that *in vivo* the intestinal epithelium is not homogenously differentiated; along the crypt villus axis there is a gradient of differentiation as immature cells migrate from the crypt base to the villus tip and become more mature^43^. This is accompanied by an increase in brush border enzyme expression. Using histological analysis of intestinal tissue, we and others have shown that brush border enzyme expression decreases from the villus tip to the crypt^44–46^. Here, we found that both scaffolds in the bioreactors and static had reduced alkaline phosphatase at the base of each villi. We also had a dramatic increase of mucus staining, which is in good correlation to data reported by other groups^20^.

Caco-2 grown in static conditions and in the bioreactors were assessed for their ability to transport glucose from the apical to basolateral membranes. We chose to perform these studies at 21 days since this was the time frame at which both bioreactor and static monolayers were in the plateau/differentiated stage of the culture. Transport in the intestine involves the use of two main glucose transporters, SGLT-1 and Glut-2^47–50^. Active transporter, SGLT-1, is located in enterocytes on the apical membrane, and Glut-2, another ATP-dependent transporter, is located on the basolateral membrane, although in hyperglycemic conditions Glut-2 has also been found to also be upregulated to the apical membrane to facilitate the transport of the excess glucose^47^. Similarly, in a recent paper using a microfluidic device, Miura *et al*. found that flow induced shear stress induced the formation of microvilli in placental epithelial cells, and in turn induced the up-regulation of Glut-1 to the apical membrane and a significant increase in glucose transporter mRNA^51^. Applying shear stress to endothelial cells, Cucullo *et al*. found significant up-regulation of Glut-1, Glut-2, Glut-3 and Glut-5 and marked increase in glucose consumption^52^. We measured active transport of glucose by the epithelial layer by feeding the cells glucose in the apical compartment and measuring glucose concentrations in the basolateral compartment at regular intervals. In addition, we blocked the Glut-2 and SGLT-1 transporters with the drugs Phlorizin and Phloretin^47^ so that we could measure the passive diffusion though the scaffold, hence any difference in glucose transfer from apical to basolateral sides after addition of the drugs represented the active transport. Caco-2 monolayers under flow conditions actively transported more glucose than the static systems. In accordance with the literature, flow conditions and the enhanced 3-D geometry facilitate the following conclusions regarding glucose transport: (i) there is an upregulation of glucose transporters, and (ii) increased shear stress may also improve absorption rates by (iii) increasing available glucose via improved luminal mixing and reduction of the unstirred layer.

Overall, this work has shown that we have created a model that may be used to more realistically simulate absorption and paracellular permeability than traditional Transwell models. In particular, the gradient of differentiation along the crypt-villus axis as a result of flow is something that is missing from the literature. However, this model is limited by the single-cell type of Caco-2, and future directions would benefit from an organotypic cell line in order to more accurately replicate the crypt region at the base of the scaffold. In addition, we only show flow in one direction, and in the native intestine there is segmentation as well as peristalsis that moves flow back and forth.

## Conclusion

We have created a device that allows for culture of Caco-2 cells on villus scaffolds. We cultivated cells in these systems for >3 weeks, and found site-specific expression profiles of cell differentiation and apoptosis along the crypt-villus axis which are more similar to *in vivo* than Transwell culture or static 3-D models. In addition, we evaluated intestinal function by measuring the rate of glucose absorption through the epithelial monolayers, finding significantly more uptake than in static systems. Importantly, levels reached are consistent with estimates for physiological conditions. We anticipate that such small intestinal bioreactors will be useful for future studies of intestinal function, including high throughput drug absorption profiling and studies of bacteria-host interactions.

## Materials and Methods

### Scaffold and Bioreactor Fabrication

Scaffolds with intestinal villus surface topography were fabricated as described previously^6^, with 10% polyethyelene-vinyl-acetate (PEVA) as the primary scaffolding material. Briefly, PEVA (10%) was dissolved in chloroform, mixed with fine sodium bicarbonate powder (400 mg/ml), and cast onto agarose molds. The scaffolds were frozen at −20 °C overnight, followed by submersion in ice cold ethanol to extract the chloroform, and warm sterile distilled water to dissolve the sodium bicarbonate, which rendered the scaffolds porous. The bioreactor was fabricated via 3-D printing in an Object30 Pro 3D-printer, using VeroClear-RGD810 as the source material. The design was modelled using CAD software (Autodesk®), and the resulting device consisted of two square pieces designed to enclose a 10 × 10 mm scaffold and form an apical and basolateral space. Flow was provided with four connectors designed for tubing, and the device was sealed four 2 mm diameter holes for screws, two sockets for o-rings. Two 0.55 mm diameter side channels were for added for TEER measurements (Fig. 1). After printing, the bioreactor was coated with a 4.5 µm layer of Parylene in a SCS Labcoter 2 Parylene Deposition System (Specialty Coating Systems, Indianapolis, Indiana) for biocompatibility. Scaffolds were sterilized in 70% EtOH, and the printed bioreactor parts were sterilized under UV. In a biosafety cabinet, scaffolds were attached to the basolateral compartments of the bioreactors, and after cell seeding the apical compartment was placed carefully on top and secured with screws. The complete device was connected to two influent and two effluent tubes. The influent tubes connected the reactors to a peristaltic pump (L/S^®^ Eight-Channel, Four-Roller Cartridge Pump System; 115/230 VAC, Vermon hills, IL), and the pump was sequentially connected to two 0.45 µm air filtered (Millipore, Tullagreen, Ireland) 900 ml influent media bottle for each apical and basolateral component of the reactors. The reactor was set to an incremental flow rate of 100 µl/min for 10 min followed by a period of stasis for 90 min. As static (non-fluidic) controls, scaffolds were incorporated into a custom insert kit with apical and basolateral sides, as described previously^5^.

### Flow Field Simulations

Computational fluid dynamic (CFD) simulations with regard to fluid flow and shear force were performed on the bioreactor model in “*Autodesk Simulation CFD 2015*” to achieve theoretical flows and shear stresses and in each direction of the bioreactor and scaffold. The assembled model used for CFD simulations was built up of to-scale molds of the scaffold, simplified reactor components, the silver TEER wire and a CAD version of the villus scaffold. The fluid assigned to the assembly model was designed to resemble Dulbecco’ Modified Eagle Medium (DMEM) at 37 °C. The fluid was assigned a density of 990 kg/m^3^ and a viscosity of 0.0078 Poise. The boundary conditions for the simulations were two inlet volume flow rates at 0.1 ml/min each at the two tube endings on one side, and 2 outlet pressure boundaries at 0 Pa at the tube endings on the opposite side of the reactor assembly. The simulations were solved with 50 iterations under laminar conditions.

### Tissue Culture

Caco-2 cells (ATCC, Manassas, VA) passage 25–35 were maintained and expanded in culture flasks in Dulbecco’s Modified Eagle Medium (DMEM) with 10% fetal bovine serum (FBS) and 1 x antibiotic-antimycotic. The cells were kept in a humidified 37 °C incubator with 5% CO_2_. Media was changed every 2–3 days and with regular passage 1–2 times a week. Prior to cell seeding, cells were removed from the flasks with 0.25% (v/v) trypsin, 0.02% ETDA Solution in 1 x PBS and adjusted to a concentration of 1 × 10^6^ cells/ml in media. The scaffolds were seeded with 50 µl cell suspension, followed by a 30 minute period without additional media to enable cell attachment. Fresh media was supplied to the bioreactor influent bottles and the static scaffolds every 2–3 days, and after 3–5 weeks the scaffolds were removed from all devices for analysis.

### TEER measurements

TEER was measured over the Caco-2 monolayers with an EVOM^2^ Epithelial Voltohmmeter with STX3 electrodes (World Precision Instruments, Sarasota, FL). The TEER in the bioreactors was measured with silver wires connecting the apical and basolateral reactor chambers to the STX3 electrode, resulting in an elongated electrode placed on each side of the scaffolds. The static control scaffolds were measured in the custom insert kits that separate the basolateral and apical space like a typical Transwell insert. Readings were blanked against cell-free scaffolds and expressed as Ohms•cm^2^ (the membrane area being determined by the diameter of the o-ring exposing a section of scaffold to media, 0.5 cm).

### Immunofluorescence

For staining of proliferation and apoptosis, we used a Click-It EdU imaging kit and a Tunel assay (Life Technologies) according to the manufacturer’s instructions. For fluorescent staining of alkaline phosphatase, scaffolds were fixed in 4% formaldehyde (in 1 x PBS) overnight at 4 °C. The formaldehyde was removed by washing with 1 x PBS, and an antigen retrieval step was performed by incubating in 10 mM sodium-citrate buffer in a vegetable steamer for 20 mins. The scaffolds were then blocked with normal donkey serum (10% in PBS with 0.3% Triton x-100) for one hour. Samples were incubated overnight with primary antibodies: rabbit anti- alkaline phosphatase (1:200 dilution, Santa Cruz). Samples were then immersed in Alexa Flour® 488 or 555 donkey anti-rabbit secondary antibodies (Life Technologies) at a 1:500 dilution. For mucus, cells were incubated for 1 hour with Alexa Flour® 555- Wheat Germ Agglutinin (WGA). Nuclei was stained with To-Pro-3®. The scaffolds were then imaged using a Zeiss LSM880 Confocal/Multiphoton Upright Microscope, with 3-D image rendering using Volocity, and image analysis with ImageJ.

### RNA extraction and qPCR

Relative mRNA levels of glucose transporters SGLT-1 and Glut-2 were determined using quantitative real time PCR, with normalization to GAPDH. Cells were removed from scaffolds using 0.25% (v/v) trypsin, 0.02% ETDA, washed by centrifugation in 1 x PBS. RNA was isolated using an RNeasy Mini Kit (Qiagen) as per the manufacturer’s instructions. On column DNAse (Qiagen) was used to remove any contaminating DNA. Next, cDNA was formed using iScript cDNA Synthesis Kit (Biorad) and 100 ng RNA. Quantitative real-time PCR was performed with SsoAdvanced Universal SYBR Green kit (Biorad). The following primers were used: SGLT-1 Forward: CTAAAGCTGATGCCCATGTTC; SGLT-1 Reverse: AGGTTGGATAGGCGATGTTG; GLUT-2 Forward: TGCTGTCTCTGTATTCCTTGTG; GLUT-2 Reverse: TGCTCACATAACTCATCCAAGAG; GAPDH Forward: ACATCGCTCAGACACCAT; GAPDH Reverse: TGTAGTTGAGGTCAATGAAGGG.

### Glucose Assay

Glucose assays were performed on bioreactors and static scaffolds after 21 days cultivation to assess the rate of active transport of glucose from the apical to the basolateral sides. Bioreactors and static scaffolds were purged/washed with glucose-free 1 x Krebs buffer (126 mM NaCl, 2.5 mM KCl, 25 mM NaHCO_3_, 1.2 mM MgCl_2_, 2.5 mM CaCl_2_) for one hour to remove any glucose from the previous media. Control (active-transport) samples were fed 2.5 mM glucose in 1 x Krebs buffer for 6 hours in the apical compartments. Passive transport samples were fed 2.5 mM glucose, 0.5 mM Phlorizin (Cayman), 1 mM Phloretin (Cayman) in 1 x Krebs buffer for 6 hours in the apical compartments. Glucose-free 1 x Krebs buffer was used in all the basolateral compartments. Samples (10 µl) were taken at time intervals from the basolateral compartments, diluted 10 fold and the concentration of glucose was assayed using Amplex® Red Glucose/Glucose Oxidase Assay Kit (Life Technologies) as per the manufacturer’s instructions.

## Electronic supplementary material

Supplemental Information
