# Supplementary material for: Microscale Bioreactors for in situ characterization of GI epithelial cell physiology
Source: Sci Rep. 2017 Oct 2;7:12515. doi: 10.1038/s41598-017-12984-2 (PMC5624909; doi:10.1038/s41598-017-12984-2)
Supplement: Supplementary file 1 — Supplemental Information [file 41598_2017_12984_MOESM1_ESM.pdf]

Microscale Bioreactors for in situ characterization of GI epithelial cell physiology: Supplementary Data

Cait M. Costello[1], Mikkel B. Phillipsen[1], Leonard M. Hartmanis[1], Marek A. Kwasnica[1], Victor Chen[1], David Hackam[2], Matthew W. Chang[3], William E. Bentley[4], John C. March[1*]

[1]Department of Biological and Environmental Engineering, Cornell University

[2]Division of Pediatric Surgery, Department of Surgery, Johns Hopkins University

[3]Department of Biochemistry, Yong Loo Lin School of Medicine, NUS

[4]Institute for Biomedical Devices, University of Maryland

*Correspondence to jcm224@cornell.edu

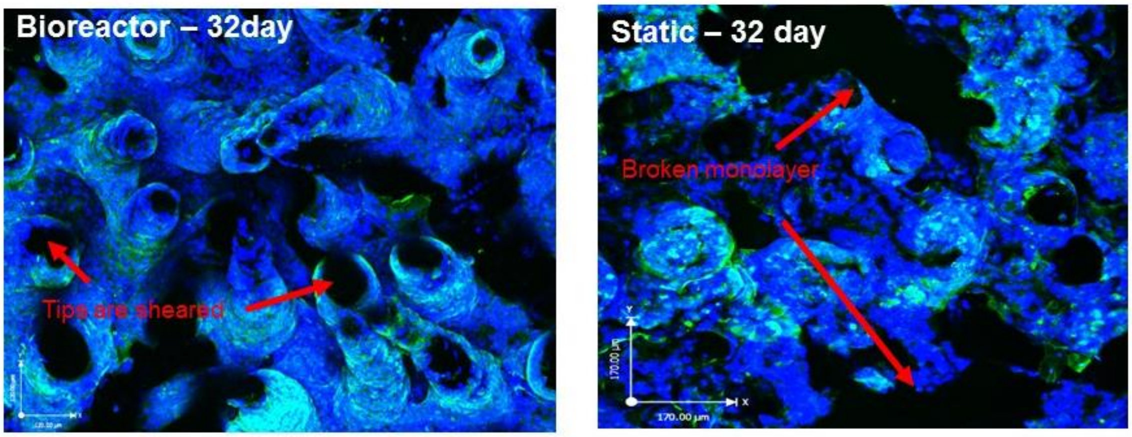

Supplementary Fig 1: 3D confocal rendering of PEVA scaffolds after growth of Caco-2 cells in the bioreactors and under static conditions for 32 days Images were taken at 10X magnification. Caco-2 were stained for alkaline phosphatase (green) and nuclei (blue). Results show that after 32 days the bioreactor scaffolds remain intact at the base, with some minimal cell lift off at the tips in the regions of high shear. In the static conditions, there is major monolayer breakdown which correlates to the decrease in TEER.

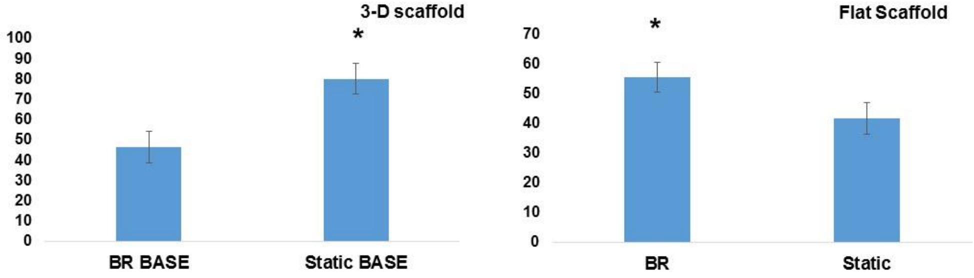

Supplementary Fig 2: Image analysis on confocal images taken from scaffolds stained for claudin-1. We used z stacks to spolit the image frames into tip and base, and measured fluorescent intensity. For the 3-D scaffold, the static conditions yielded greater fluorescence intensity at the base, whereas in the flast scaffold, the bioreactor had higher TEER.

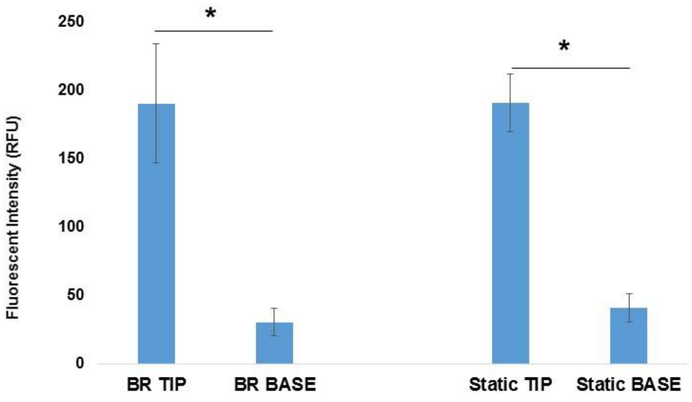

Supplementary Fig 3: Image analysis on confocal images taken from scaffolds stained for alkaline phosphatase. We counted 10 villi for each sample along the xz axis, and measured fluorescent intensity at the tip and base. Both scaffolds had significantly higher alkaline phosphatase at the tips than at the base according to unpaired t-test. The static scaffolds has slightly reduced alkaline phosphatase at the base compared to static, however this was not significant.

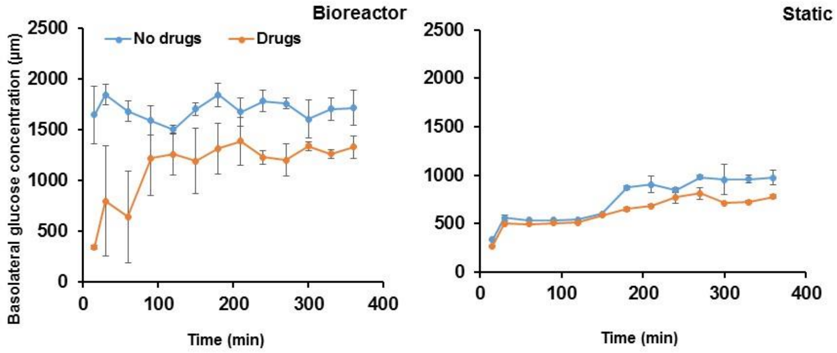

Supplementary Fig 4: Glucose assay raw data for bioreactors and scaffolds grown under static conditions. Glucose was fed apically and then sampled basolaterally at time intervals and measured using an Amplex red glucose assay. Four different scenarios were tested: i) Bioreactor just glucose/no drugs ii) Bioreactor with glucose and drugs (phlorizin and phloretin) iii) Static just glucose/no drugs iv) Static with glucose and drugs. In the graphs pictured, glucose between the blue and red lines is actively transported, and below the red lines is passive. Results show that for both bioreactor and static conditions there is passive diffusion of glucose through the scaffold, however the bioreactor has much more active transport. The glucose from the 'drugs' samples was subtracted from the 'no drugs' samples to calculate the rate of active transport, as shown in **Fig 6**.
